# Ethyl 1-(2-bromo­eth­yl)-3-(4-meth­oxy­phen­yl)-1*H*-pyrazole-5-carboxyl­ate

**DOI:** 10.1107/S1600536812032370

**Published:** 2012-07-21

**Authors:** Ling Yin, Yi Wang, Ying-Ying Wang, Jian-Wu Wang

**Affiliations:** a Department of Chemistry and Chemical Engineering, Jining University, Qufu 273155, People’s Republic of China; b School of Chemistry and Chemical Engineering, Shandong University, Jinan 250100, People’s Republic of China

## Abstract

In the title compound, C_15_H_17_BrN_2_O_3_, the dihedral angle between the benzene and pyrazole rings is 5.63 (2)°. The crystal packing is stabilized by weak π–π stacking inter­actions [centroid–centroid distance = 3.927 (5) Å] and inter­molecular C—H⋯O and C—H⋯Br hydrogen bonds.

## Related literature

For the biological activity of pyrazole compounds, see: Nagwa *et al.* (2012 ▶); Fahmy *et al.* (2012 ▶); Wang *et al.* (2011 ▶). For related structures, see: Dong *et al.* (2007 ▶); Hao *et al.* (2012 ▶).
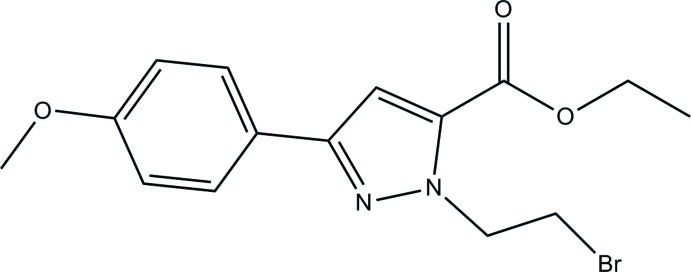


## Experimental

### 

#### Crystal data

C_15_H_17_BrN_2_O_3_
*M* *_r_* = 353.22
Monoclinic, 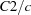

*a* = 24.691 (7) Å
*b* = 6.7678 (17) Å
*c* = 17.884 (5) Å
β = 97.184 (5)°
*V* = 2965.1 (13) Å^3^
*Z* = 8
Mo *K*α radiation
μ = 2.78 mm^−1^
*T* = 113 K
0.20 × 0.18 × 0.14 mm

#### Data collection

Rigaku Saturn CCD area-detector diffractometer
Absorption correction: multi-scan (*CrystalClear-SM Expert*; Rigaku/MSC, 2009 ▶) *T*_min_ = 0.606, *T*_max_ = 0.697
13190 measured reflections
3501 independent reflections
2684 reflections with *I* > 2σ(*I*)
*R*_int_ = 0.033

#### Refinement

*R*[*F* ^2^ > 2σ(*F* ^2^)] = 0.026
*wR*(*F* ^2^) = 0.062
*S* = 1.02
3501 reflections
192 parameters
H-atom parameters constrained
Δρ_max_ = 0.61 e Å^−3^
Δρ_min_ = −0.33 e Å^−3^

### 

Data collection: *CrystalClear-SM Expert* (Rigaku/MSC, 2009 ▶); cell refinement: *CrystalClear-SM Expert*; data reduction: *CrystalClear-SM Expert*; program(s) used to solve structure: *SHELXS97* (Sheldrick, 2008 ▶); program(s) used to refine structure: *SHELXL97* (Sheldrick, 2008 ▶); molecular graphics: *SHELXTL* (Sheldrick, 2008 ▶); software used to prepare material for publication: *SHELXTL*.

## Supplementary Material

Crystal structure: contains datablock(s) I, global. DOI: 10.1107/S1600536812032370/rz2789sup1.cif

Structure factors: contains datablock(s) I. DOI: 10.1107/S1600536812032370/rz2789Isup2.hkl

Supplementary material file. DOI: 10.1107/S1600536812032370/rz2789Isup3.cml

Additional supplementary materials:  crystallographic information; 3D view; checkCIF report

## Figures and Tables

**Table 1:** Hydrogen-bond geometry (Å, °)

*D*—H⋯*A*	*D*—H	H⋯*A*	*D*⋯*A*	*D*—H⋯*A*
C7—H7⋯Br1^i^	0.95	2.93	3.6791 (18)	137
C15—H15*B*⋯O1^ii^	0.99	2.56	3.288 (2)	130

Symmetry codes: (i) 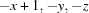; (ii) 
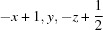
.

**Fig. 1.**
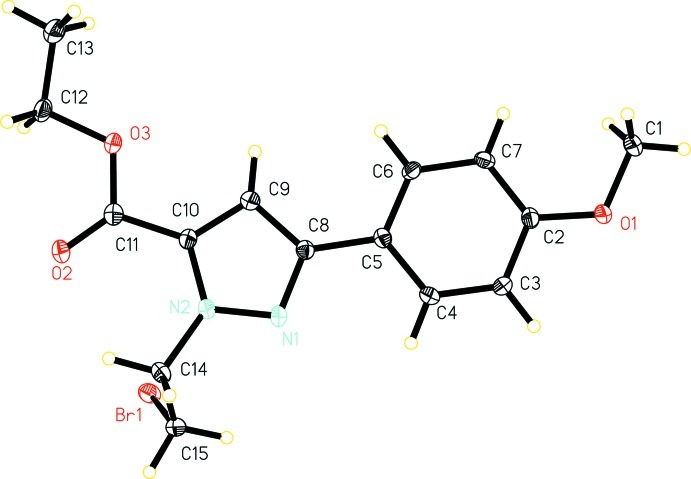
The molecular structure of the title compound, with displacement ellipsoids drawn at the 40% probability level.

## supplementary crystallographic information

### Comment

Pyrazole compounds are associated with a wide spectrum of biological activities (Nagwa *et al.*, 2012; Fahmy *et al.*, 2012). The title compound is a key intermediate during the synthesis of our own Lp-PLA_2_ inhibitors (Wang *et al.*, 2011).

In title compound (Fig. 1) the dihedral angle between the benzene ring (C2—C7) and the pyrazole ring (N1/N2/C8—C10) is 5.63 (2) °. All bond lengths and angles are normal and in a good agreement with those reported previously for related compounds (Dong *et al.*, 2007; Hao *et al.*, 2012). The crystal packing is stabilized by weak π–π stacking interactions [*Cg*1···*Cg*2^i^ = 3.697 (9) Å, *Cg*1 and *Cg*2 are the centroids of the benzene and pyrazole ring, respectively; symmetry code: (i) -x, 2-y, 1-z] and weak intermolecular C—H···O and C—H···Br hydrogen interactions (Table 1).

### Experimental

A mixture of of ethyl 3-(4-methoxyphenyl)-1*H*-pyrazole-5-carboxylate (2.46 g, 10 mmol), 1,2-dibromoethane (3.76 g, 20 mmol), K_2_CO_3_ (5.53 g, 40 mmol) in dried acetonitrile (30 ml) was refluxed overnight. On cooling, the reaction mixture was filtered and poured into 200 ml of brine. The resulting mixture was extracted with dichloromethane (3 × 50 ml), and the combined extracts were washed with saturated brine, dried over Na_2_SO_4_ and evaporated on a rotary evaporator to afford the crude product as brown solid, which was purified by column chromatography to yield the pure product as colourless crystals (yield 88%). Single crystals suitable for single-crystal X-ray diffraction were obtained by slow evaporation at room temperature of a solution of the title compound in dichloromethane/hexane (1:5 *v*/*v*). M.p.: 361–363 K.

### Refinement

All H atoms were found in a difference Fourier map and refined using a riding model, with C–H = 0.95–0.99 Å and with *U*_iso_(H) = 1.2 *U*_eq_(C) or 1.5*U*_eq_(C) for methyl H atoms.

### Crystal data

**Table:**

C_15_H_17_BrN_2_O_3_	*F*(000) = 1440
*M**_r_* = 353.22	*D*_x_ = 1.583 Mg m^−^^3^
Monoclinic, *C*2/*c*	Mo *K*α radiation, λ = 0.71073 Å
Hall symbol: -C 2yc	Cell parameters from 5090 reflections
*a* = 24.691 (7) Å	θ = 1.9–27.9°
*b* = 6.7678 (17) Å	µ = 2.78 mm^−^^1^
*c* = 17.884 (5) Å	*T* = 113 K
β = 97.184 (5)°	Prism, colourless
*V* = 2965.1 (13) Å^3^	0.20 × 0.18 × 0.14 mm
*Z* = 8	

### Data collection

**Table:**

Rigaku Saturn CCD area-detector diffractometer	3501 independent reflections
Radiation source: rotating anode	2684 reflections with *I* > 2σ(*I*)
Multilayer monochromator	*R*_int_ = 0.033
Detector resolution: 14.63 pixels mm^-1^	θ_max_ = 27.9°, θ_min_ = 2.3°
ω and φ scans	*h* = −32→30
Absorption correction: multi-scan (*CrystalClear-SM Expert*; Rigaku/MSC, 2009)	*k* = −8→8
*T*_min_ = 0.606, *T*_max_ = 0.697	*l* = −23→20
13190 measured reflections	

### Refinement

**Table:**

Refinement on *F*^2^	Primary atom site location: structure-invariant direct methods
Least-squares matrix: full	Secondary atom site location: difference Fourier map
*R*[*F*^2^ > 2σ(*F*^2^)] = 0.026	Hydrogen site location: inferred from neighbouring sites
*wR*(*F*^2^) = 0.062	H-atom parameters constrained
*S* = 1.02	*w* = 1/[σ^2^(*F*_o_^2^) + (0.0311*P*)^2^] where *P* = (*F*_o_^2^ + 2*F*_c_^2^)/3
3501 reflections	(Δ/σ)_max_ = 0.002
192 parameters	Δρ_max_ = 0.61 e Å^−^^3^
0 restraints	Δρ_min_ = −0.33 e Å^−^^3^

### Special details

**Table:**

Geometry. All e.s.d.'s (except the e.s.d. in the dihedral angle between two l.s. planes) are estimated using the full covariance matrix. The cell e.s.d.'s are taken into account individually in the estimation of e.s.d.'s in distances, angles and torsion angles; correlations between e.s.d.'s in cell parameters are only used when they are defined by crystal symmetry. An approximate (isotropic) treatment of cell e.s.d.'s is used for estimating e.s.d.'s involving l.s. planes.
Refinement. Refinement of *F*^2^ against ALL reflections. The weighted *R*-factor *wR* and goodness of fit *S* are based on *F*^2^, conventional *R*-factors *R* are based on *F*, with *F* set to zero for negative *F*^2^. The threshold expression of *F*^2^ > σ(*F*^2^) is used only for calculating *R*-factors(gt) *etc*. and is not relevant to the choice of reflections for refinement. *R*-factors based on *F*^2^ are statistically about twice as large as those based on *F*, and *R*- factors based on ALL data will be even larger.

### Fractional atomic coordinates and isotropic or equivalent isotropic displacement parameters (Å^2^)

**Table:**

	*x*	*y*	*z*	*U*_iso_*/*U*_eq_	
Br1	0.300383 (7)	−0.03804 (3)	0.042640 (10)	0.02615 (7)	
O1	0.67705 (4)	0.29412 (19)	0.23584 (6)	0.0228 (3)	
O2	0.32875 (5)	0.23696 (19)	−0.13898 (7)	0.0238 (3)	
O3	0.41059 (5)	0.20467 (18)	−0.18169 (6)	0.0207 (3)	
N1	0.43218 (5)	0.3178 (2)	0.06953 (8)	0.0196 (3)	
N2	0.39195 (5)	0.3026 (2)	0.01120 (8)	0.0190 (3)	
C1	0.72814 (7)	0.2707 (3)	0.20691 (11)	0.0295 (5)	
H1A	0.7284	0.1445	0.1800	0.044*	
H1B	0.7579	0.2722	0.2487	0.044*	
H1C	0.7332	0.3792	0.1722	0.044*	
C2	0.63072 (6)	0.2885 (3)	0.18455 (10)	0.0177 (4)	
C3	0.58195 (7)	0.3140 (3)	0.21405 (10)	0.0215 (4)	
H3	0.5821	0.3351	0.2666	0.026*	
C4	0.53298 (7)	0.3087 (3)	0.16717 (10)	0.0212 (4)	
H4	0.4998	0.3271	0.1881	0.025*	
C5	0.53125 (6)	0.2770 (2)	0.08977 (9)	0.0162 (4)	
C6	0.58044 (7)	0.2519 (3)	0.06120 (10)	0.0177 (4)	
H6	0.5803	0.2300	0.0087	0.021*	
C7	0.63016 (7)	0.2582 (3)	0.10763 (10)	0.0183 (4)	
H7	0.6634	0.2418	0.0868	0.022*	
C8	0.47850 (6)	0.2734 (2)	0.04107 (9)	0.0162 (4)	
C9	0.46766 (7)	0.2326 (2)	−0.03645 (9)	0.0174 (4)	
H9	0.4933	0.1983	−0.0697	0.021*	
C10	0.41209 (7)	0.2527 (2)	−0.05401 (9)	0.0168 (4)	
C11	0.37810 (7)	0.2314 (3)	−0.12779 (10)	0.0185 (4)	
C12	0.38331 (7)	0.1924 (3)	−0.25861 (9)	0.0231 (4)	
H12A	0.3609	0.3120	−0.2712	0.028*	
H12B	0.3591	0.0753	−0.2645	0.028*	
C13	0.42739 (7)	0.1759 (3)	−0.30925 (10)	0.0291 (5)	
H13A	0.4507	0.2935	−0.3033	0.044*	
H13B	0.4107	0.1654	−0.3618	0.044*	
H13C	0.4495	0.0580	−0.2956	0.044*	
C14	0.33701 (7)	0.3614 (3)	0.02402 (10)	0.0221 (4)	
H14A	0.3127	0.3563	−0.0244	0.026*	
H14B	0.3379	0.4997	0.0422	0.026*	
C15	0.31375 (7)	0.2311 (3)	0.08072 (10)	0.0242 (4)	
H15A	0.2790	0.2887	0.0929	0.029*	
H15B	0.3395	0.2267	0.1278	0.029*	

### Atomic displacement parameters (Å^2^)

**Table:**

	*U*^11^	*U*^22^	*U*^33^	*U*^12^	*U*^13^	*U*^23^
Br1	0.02115 (10)	0.03091 (12)	0.02741 (12)	0.00178 (8)	0.00701 (8)	0.00177 (8)
O1	0.0133 (6)	0.0390 (8)	0.0157 (7)	−0.0006 (5)	0.0001 (5)	0.0010 (5)
O2	0.0158 (6)	0.0325 (8)	0.0219 (7)	−0.0010 (5)	−0.0023 (5)	0.0001 (5)
O3	0.0177 (6)	0.0287 (8)	0.0148 (7)	−0.0011 (5)	−0.0011 (5)	−0.0004 (5)
N1	0.0158 (7)	0.0257 (9)	0.0163 (8)	0.0004 (6)	−0.0020 (6)	0.0001 (6)
N2	0.0128 (7)	0.0258 (9)	0.0176 (8)	0.0006 (6)	−0.0010 (6)	0.0005 (6)
C1	0.0138 (9)	0.0494 (14)	0.0247 (11)	−0.0010 (8)	−0.0004 (8)	0.0007 (9)
C2	0.0154 (8)	0.0191 (9)	0.0178 (9)	−0.0018 (7)	−0.0006 (7)	0.0024 (7)
C3	0.0190 (9)	0.0317 (11)	0.0139 (9)	−0.0002 (7)	0.0024 (7)	−0.0020 (7)
C4	0.0145 (8)	0.0304 (11)	0.0195 (10)	−0.0004 (7)	0.0049 (7)	0.0000 (8)
C5	0.0147 (8)	0.0158 (9)	0.0179 (9)	−0.0008 (6)	0.0008 (7)	0.0011 (7)
C6	0.0198 (8)	0.0208 (10)	0.0127 (9)	−0.0015 (7)	0.0027 (7)	−0.0006 (7)
C7	0.0144 (8)	0.0223 (10)	0.0189 (9)	0.0009 (7)	0.0057 (7)	0.0007 (7)
C8	0.0155 (8)	0.0160 (9)	0.0168 (9)	−0.0012 (6)	0.0013 (7)	0.0014 (7)
C9	0.0172 (8)	0.0173 (9)	0.0179 (9)	0.0000 (7)	0.0027 (7)	0.0001 (7)
C10	0.0173 (8)	0.0174 (9)	0.0153 (9)	−0.0010 (7)	0.0000 (7)	0.0002 (7)
C11	0.0193 (9)	0.0161 (9)	0.0196 (10)	−0.0014 (7)	0.0000 (7)	0.0015 (7)
C12	0.0225 (9)	0.0291 (11)	0.0161 (9)	−0.0032 (8)	−0.0033 (7)	−0.0001 (7)
C13	0.0281 (10)	0.0385 (13)	0.0198 (10)	−0.0039 (9)	−0.0004 (8)	−0.0006 (8)
C14	0.0146 (8)	0.0282 (11)	0.0231 (10)	0.0038 (7)	0.0011 (7)	−0.0027 (8)
C15	0.0181 (9)	0.0363 (12)	0.0185 (10)	0.0004 (8)	0.0037 (8)	−0.0050 (8)

### Geometric parameters (Å, º)

**Table:**

Br1—C15	1.9581 (19)	C5—C8	1.474 (2)
O1—C2	1.3746 (18)	C6—C7	1.394 (2)
O1—C1	1.431 (2)	C6—H6	0.9500
O2—C11	1.2103 (19)	C7—H7	0.9500
O3—C11	1.341 (2)	C8—C9	1.406 (2)
O3—C12	1.456 (2)	C9—C10	1.375 (2)
N1—C8	1.343 (2)	C9—H9	0.9500
N1—N2	1.3515 (18)	C10—C11	1.480 (2)
N2—C10	1.366 (2)	C12—C13	1.505 (2)
N2—C14	1.459 (2)	C12—H12A	0.9900
C1—H1A	0.9800	C12—H12B	0.9900
C1—H1B	0.9800	C13—H13A	0.9800
C1—H1C	0.9800	C13—H13B	0.9800
C2—C3	1.384 (2)	C13—H13C	0.9800
C2—C7	1.389 (2)	C14—C15	1.511 (2)
C3—C4	1.383 (2)	C14—H14A	0.9900
C3—H3	0.9500	C14—H14B	0.9900
C4—C5	1.396 (2)	C15—H15A	0.9900
C4—H4	0.9500	C15—H15B	0.9900
C5—C6	1.386 (2)		
			
C2—O1—C1	116.96 (13)	C10—C9—C8	105.49 (15)
C11—O3—C12	116.03 (13)	C10—C9—H9	127.3
C8—N1—N2	105.62 (13)	C8—C9—H9	127.3
N1—N2—C10	111.51 (13)	N2—C10—C9	106.80 (14)
N1—N2—C14	117.78 (13)	N2—C10—C11	123.97 (15)
C10—N2—C14	130.28 (14)	C9—C10—C11	129.22 (16)
O1—C1—H1A	109.5	O2—C11—O3	124.45 (16)
O1—C1—H1B	109.5	O2—C11—C10	126.26 (17)
H1A—C1—H1B	109.5	O3—C11—C10	109.29 (14)
O1—C1—H1C	109.5	O3—C12—C13	106.78 (14)
H1A—C1—H1C	109.5	O3—C12—H12A	110.4
H1B—C1—H1C	109.5	C13—C12—H12A	110.4
O1—C2—C3	115.67 (15)	O3—C12—H12B	110.4
O1—C2—C7	124.70 (15)	C13—C12—H12B	110.4
C3—C2—C7	119.63 (15)	H12A—C12—H12B	108.6
C4—C3—C2	120.12 (16)	C12—C13—H13A	109.5
C4—C3—H3	119.9	C12—C13—H13B	109.5
C2—C3—H3	119.9	H13A—C13—H13B	109.5
C3—C4—C5	121.41 (16)	C12—C13—H13C	109.5
C3—C4—H4	119.3	H13A—C13—H13C	109.5
C5—C4—H4	119.3	H13B—C13—H13C	109.5
C6—C5—C4	117.70 (15)	N2—C14—C15	112.65 (15)
C6—C5—C8	122.05 (15)	N2—C14—H14A	109.1
C4—C5—C8	120.25 (15)	C15—C14—H14A	109.1
C5—C6—C7	121.58 (16)	N2—C14—H14B	109.1
C5—C6—H6	119.2	C15—C14—H14B	109.1
C7—C6—H6	119.2	H14A—C14—H14B	107.8
C2—C7—C6	119.56 (15)	C14—C15—Br1	111.80 (12)
C2—C7—H7	120.2	C14—C15—H15A	109.3
C6—C7—H7	120.2	Br1—C15—H15A	109.3
N1—C8—C9	110.58 (15)	C14—C15—H15B	109.3
N1—C8—C5	120.26 (15)	Br1—C15—H15B	109.3
C9—C8—C5	129.14 (15)	H15A—C15—H15B	107.9
			
C8—N1—N2—C10	−1.04 (18)	C4—C5—C8—C9	−175.81 (17)
C8—N1—N2—C14	−174.27 (15)	N1—C8—C9—C10	−0.5 (2)
C1—O1—C2—C3	−179.24 (16)	C5—C8—C9—C10	−178.89 (16)
C1—O1—C2—C7	1.2 (2)	N1—N2—C10—C9	0.77 (19)
O1—C2—C3—C4	−179.36 (16)	C14—N2—C10—C9	172.91 (17)
C7—C2—C3—C4	0.2 (3)	N1—N2—C10—C11	−178.04 (15)
C2—C3—C4—C5	0.3 (3)	C14—N2—C10—C11	−5.9 (3)
C3—C4—C5—C6	−0.4 (3)	C8—C9—C10—N2	−0.18 (19)
C3—C4—C5—C8	−179.62 (16)	C8—C9—C10—C11	178.54 (17)
C4—C5—C6—C7	−0.1 (3)	C12—O3—C11—O2	3.3 (2)
C8—C5—C6—C7	179.16 (16)	C12—O3—C11—C10	−176.61 (14)
O1—C2—C7—C6	178.88 (15)	N2—C10—C11—O2	−7.2 (3)
C3—C2—C7—C6	−0.6 (3)	C9—C10—C11—O2	174.33 (19)
C5—C6—C7—C2	0.6 (3)	N2—C10—C11—O3	172.76 (16)
N2—N1—C8—C9	0.91 (19)	C9—C10—C11—O3	−5.8 (2)
N2—N1—C8—C5	179.50 (14)	C11—O3—C12—C13	176.44 (15)
C6—C5—C8—N1	−173.34 (16)	N1—N2—C14—C15	−64.7 (2)
C4—C5—C8—N1	5.9 (2)	C10—N2—C14—C15	123.56 (19)
C6—C5—C8—C9	5.0 (3)	N2—C14—C15—Br1	−67.21 (17)

### Hydrogen-bond geometry (Å, º)

**Table:**

*D*—H···*A*	*D*—H	H···*A*	*D*···*A*	*D*—H···*A*
C7—H7···Br1^i^	0.95	2.93	3.6791 (18)	137
C15—H15*B*···O1^ii^	0.99	2.56	3.288 (2)	130

Symmetry codes: (i) −*x*+1, −*y*, −*z*; (ii) −*x*+1, *y*, −*z*+1/2.

## References

[bb1] Dong, W.-L., Ge, Y.-Q., Xia, Y. & Zhao, B.-X. (2007). *Acta Cryst.* E**63**, o4701.

[bb2] Fahmy, H. H., Khalifa, N. M., Nossier, E. S., Abdalla, M. M. & Ismai, M. M. (2012). *Acta Pol. Pharm.* **69**, 411–421. 22594255

[bb3] Hao, B.-Q., Xu, W.-R., Meng, F.-C. & Duan, G.-Y. (2012). *Acta Cryst.* E**68**, o877. 10.1107/S1600536812007428 PMC3297926 22412729

[bb4] Nagwa, M., Gawad, A. E., Georgey, H. H., Ibrahim, N. A., Amin, N. H. & Abdelsalam, R. M. (2012). *Arch. Pharm. Res.* **35**, 807–821. 10.1007/s12272-012-0507-y 22644849

[bb5] Rigaku/MSC (2009). *CrystalClear-SM Expert* Rigaku/MSC, The Woodlands, Texas, USA.

[bb6] Sheldrick, G. M. (2008). *Acta Cryst.* A**64**, 112–122. 10.1107/S0108767307043930 18156677

[bb7] Wang, Y., Xu, W. R., Shao, H., Xie, Y. F. & Wang, J. W. (2011). *Chin. J. Chem.* **29**, 2039–2048.

